# Delirium Tremens

**Published:** 1854-12

**Authors:** 


Delirium Tremens.—A boy, calling on a doctor to visit his father, who had delirium tremens, and not rightly recollecting the name of the disease, called it the *devil's trembles*, thus making bad Latin, but very good English.

